# Letter to the Editor

**Published:** 1910-09

**Authors:** H. M. Folkes


### Letter to the Editor.

If Osteoquacks in other places pursue the methods that are in vogue among some of the practitioners of that cult in this community, it were easy to understand the popularity of this fad among certain of our women folks.

There have been in this city, to my personal knowledge, quite a dozen miscarriages brought on by this treatment and such addenda as vaginal packing, together at times with probable instrumental interference.

From three of these cases of which I have personal knowledge, the mere rubbing alone has apparently sufficed to bring on abortion, two of these patients being primiparae, and certainly in two instances the miscarriages were undesired, as the women were very anxious for children.

Other physicians having like experience should promptly make report thereof, for while this feature of osteoquackery will doubtless be popular among women who have no desire to bear children, yet a great many women are anxious to become mothers and would certainly not jeopardize their chances of maternity by subjecting themselves to such treatment.—*H. M. Folkes, in Mississippi Medical Monthly.*

